# Gap junction proteins and their role in spinal cord injury

**DOI:** 10.3389/fnmol.2014.00102

**Published:** 2015-01-06

**Authors:** Ryan S. Tonkin, Yilin Mao, Simon J. O’Carroll, Louise F. B. Nicholson, Colin R. Green, Catherine A. Gorrie, Gila Moalem-Taylor

**Affiliations:** ^1^School of Medical Sciences, Faculty of Medicine, University of New South WalesSydney, NSW, Australia; ^2^School of Medical and Molecular Bioscience, Faculty of Science, University of TechnologySydney, NSW, Australia; ^3^Department of Anatomy with Radiology and Centre for Brain Research, Faculty of Medical and Health Sciences, University of AucklandAuckland, New Zealand; ^4^Department of Ophthalmology, Faculty of Medical and Health Sciences, University of AucklandAuckland, New Zealand

**Keywords:** spinal cord injury, gap junctions, hemichannels, connexins, neuropathic pain

## Abstract

Gap junctions are specialized intercellular communication channels that are formed by two hexameric connexin hemichannels, one provided by each of the two adjacent cells. Gap junctions and hemichannels play an important role in regulating cellular metabolism, signaling, and functions in both normal and pathological conditions. Following spinal cord injury (SCI), there is damage and disturbance to the neuronal elements of the spinal cord including severing of axon tracts and rapid cell death. The initial mechanical disruption is followed by multiple secondary cascades that cause further tissue loss and dysfunction. Recent studies have implicated connexin proteins as playing a critical role in the secondary phase of SCI by propagating death signals through extensive glial networks. In this review, we bring together past and current studies to outline the distribution, changes and roles of various connexins found in neurons and glial cells, before and in response to SCI. We discuss the contribution of pathologically activated connexin proteins, in particular connexin 43, to functional recovery and neuropathic pain, as well as providing an update on potential connexin specific pharmacological agents to treat SCI.

## INTRODUCTION

**FIGURE 1:**
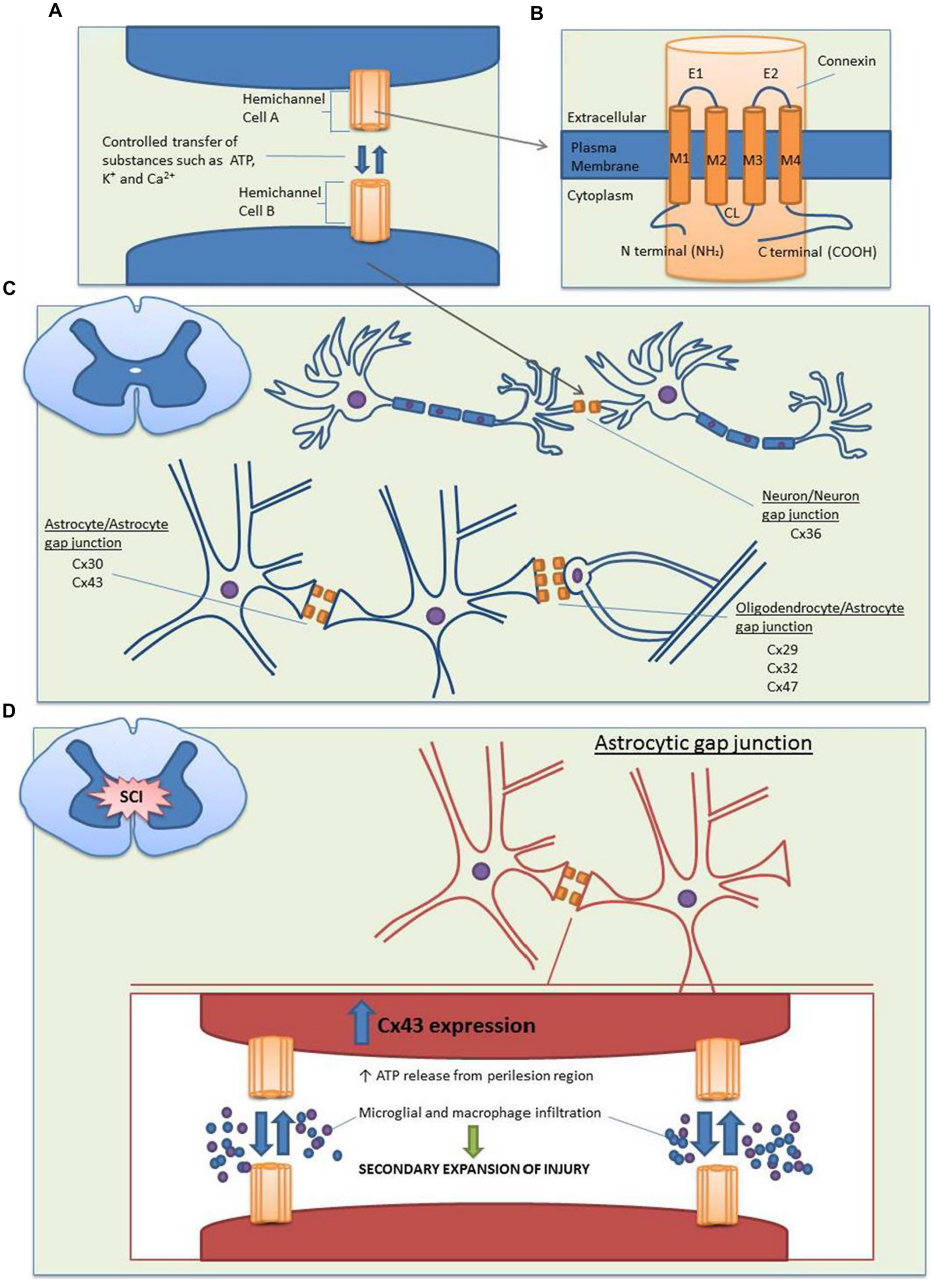
**Structure and distribution of the main connexin proteins in the spinal cord before and after SCI. (A)** Gap junction channels are produced by the conjoining of two hemichannels, with one hemichannel provided by each cell. Compared to the closed state (∼1.8 nm), opened channels (∼2.5 nm) allow the transfer of substances less than 1 kDa between the cytoplasms of two cells, including nutrients, metabolites, K^+^, ATP, cAMP, and Ca^2+^. **(B)** Each hemichannel is composed of six individual connexin proteins, which contain four transmembrane domains. The cytoplasmic loop (CL), amino (N), and carboxy (C) terminals are located on the cytoplasmic side, with multiple regulatory phosphorylation sites located on the C termini. There are two extracellular loops (E1 and E2) which contain three highly conserved cysteine bonds that play a role in the docking of hemichannels from opposing cells via intramolecular cysteine/cysteine bonds. **(C)** In the spinal cord, there is a large distribution of connexins. Neuron/neuron gap junctions contain Cx36, astrocyte/astrocyte junctions contain Cx30 and Cx43 and oligodendrocyte/astrocyte gap junctions contain Cx29, Cx32, and Cx47 with the major heterotypic channels composed of Cx43Cx47 and Cx30Cx32. **(D)** Following SCI, Cx43 expression is increased in astrocytes. This causes an uncontrolled release of the small molecule ATP from the perilesion region. ATP release leads to the activation of a destructive inflammatory response including recruitment of microglia and macrophages to the site of injury, which causes an increased secondary expansion of the lesion.

Gap junctions are specialized intercellular connections that allow direct electrical and metabolic communication between two adjacent cells; they are therefore vital to many physiological processes. By controlling the movement of amino acids, second messengers, ions, and other metabolites, gap junctions are able to coordinate cellular signaling and propagate electrical signals (Hertzberg et al., 1981; Söhl et al., 2005). In vertebrates, gap junctions are formed by two hemichannels (or connexons), one provided by each of two cells. Each hemichannel consists of six oligomerized connexin proteins (abbreviated as Cx), which can be either homomeric or heteromeric (two or more different connexins in a connexon) and form homotypic or heterotypic (different connexons) gap junction channels (Orthmann-Murphy et al., 2008). Connexins are named according to their estimated molecular weights and have been shown to be expressed in almost all mammalian tissues (Söhl and Willecke, 2003; Söhl et al., 2005). The structure of these connexin proteins is described in **Figures 1A,B**.

While two hemichannels form gap junctions that directly connect the interiors of two adjacent cells without facing the external milieu, increasing evidence indicates that unopposed hemichannels can open to the extracellular space in response to various physiological and pathological stimuli (Bennett et al., 2003). In such contexts, connexin hemichannels may become activated in response to various intracellular signals including changes in pH, phosphorylation status, extracellular signals such as a low Ca^2+^ environment, and mechanical, oxidative, metabolic, and ischemic stresses (Contreras et al., 2002; Retamal et al., 2007; Shintani-Ishida et al., 2007). Indeed, studies using dye uptake measurements, recording of hemichannel activity in single cells, and pharmacological blockade of hemichannels in transfected cells have demonstrated that connexins form unopposed hemichannels that can open to the exterior (Contreras et al., 2003; Li et al., 2004). Opened hemichannels lead to the release of molecules such as potassium, ATP, and glutamate into the extracellular space, that under physiological conditions may modulate neuronal activity and under pathological conditions may ultimately induce cell death (Evans et al., 2006; Chever et al., 2014). In addition, gap junctional communication can be likewise regulated by numerous signals, such as connexin phosphorylation and post-translational modification at the C-terminal tail, intracellular acidification, intracellular pH, and inflammatory mediators (Lampe and Lau, 2000; Faustmann et al., 2003; Hinkerohe et al., 2005; Haghikia et al., 2008; Iyyathurai et al., 2013).

In this review, we discuss the expression and function of gap junction/hemichannel proteins in the intact and injured spinal cord, their role in neuropathic pain following spinal cord injury (SCI), and the therapeutic implications.

## DISTRIBUTION AND ROLES OF CONNEXINS IN THE SPINAL CORD

In the normal functioning central nervous system (CNS), gap junctions are formed by fully docked connexons, which play a vital physiological role in the development of communicative pathways in glial and neuronal cells. Studies examining the distribution and functional role of connexins in the spinal cord indicate that connexin-formed gap junctions undergo cell-to-cell coupling between neurons, adjacent astrocytes, and astrocytes/oligodendrocytes (**Figure 1C**; Chang et al., 1999; Chang and Balice-Gordon, 2000; Rash et al., 2000; Kleopa et al., 2004; Lee et al., 2005; Orthmann-Murphy et al., 2008).

In neurons, gap junctional coupling is widespread during neural development but declines during the first postnatal week. Gap junctional communication in the developing spinal cord is vital in synchronizing neuronal cells and providing electrical synapses, which act as support frameworks for neuronal activity prior to the development of chemical synapses (Chang et al., 1999; Kiehn et al., 2000; Altevogt et al., 2002; Kiehn and Tresch, 2002; Wilson et al., 2007). Developing motor neurons in the spinal cord express Cx36, Cx37, Cx40, Cx43, and Cx45 mRNA. While electrical and dye coupling decrease at about 1 week postnatal along with Cx40 and Cx45 expression, the remaining connexins persist into adulthood (Chang et al., 1999; Chang and Balice-Gordon, 2000). Although earlier studies by Chang et al. (1999) were unable to demonstrate Cx36 and Cx37 protein by immunohistochemistry, more recent studies using sensitive techniques such as *in situ* hybridization and freeze-fracture replica immunogold labeling (FRIL) were able to detect Cx36 mRNA and protein, respectively. Cx36 mRNA was found to be confined to the cytoplasm of spinal neurons but was more strongly expressed in developing neurons compared to adult rodent spinal cord neurons (Lee et al., 2005). A recent study using transgenic mice expressing Cx36 protein tagged with enhanced green florescent protein, showed a substantial number of florescent clusters in the white matter of the spinal cord providing further evidence for the presence of Cx36 in the adult spinal cord (Meyer et al., 2014). Using FRIL, there was no evidence for the presence of Cx26 in neuronal gap junctions in the perinatal or adult spinal cord (Nagy et al., 2001). While it may be possible that there are as yet unidentified connexins in neuronal gap junctions, more recent studies have implicated Cx36 as the major connexin in mediating electrical synapses in neurons of the spinal cord (Bautista et al., 2014). In adults, neuronal gap junction channels are proposed to contribute to a number of different cognitive processes such as perception, memory, and learning (Buzsáki and Chrobak, 1995; Fricker and Miles, 2001). These gap junction channels are able to sharpen neuronal activity by enhancing the efficacy and precision of synchronous oscillatory activity in neurons (Hormuzdi et al., 2004; Gibson et al., 2005).

In astrocytes, Cx43 and Cx30 are abundantly expressed and are found densely populated around the ependymal and leptomeningeal membranes of the neonatal rodent spinal cord, roughly 4 weeks postnatal (Dahl et al., 1996; Kunzelmann et al., 1999; Lee et al., 2005). It has also been shown using FRIL analysis that leptomeningeal cells in the rats midthoracic spinal cord are highly labeled for Cx26, and that most astrocyte gap junctions in the parenchyma of adult spinal cord are labeled for both Cx26 and Cx30 or Cx26 alone (Nagy et al., 2001). However, recent evidence has suggested a degree of uncertainty over the presence of Cx26 in astrocytes. In postnatal day 4 rats, regions of spinal leptomeningeal cells were found to be largely unlabeled for Cx26 using immunohistochemistry (Nagy et al., 2001). While it is possible that this result reflects the low labeling efficiency for Cx26 at postnatal day 4, recent evidence has shown that the Cx26 antibody may cross-react with Cx30 (Altevogt and Paul, 2004; Orthmann-Murphy et al., 2008). Furthermore, a study using mice with genetically altered Cx26 allele that allows visualization of Cx26 expression has shown that in both the embryonic and mature CNS, Cx26 was restricted to meningeal cells and could not be detected by either neurons or glia, including astrocytes (Filippov et al., 2003). The importance of these astrocytic connexins to normal physiology appears to be linked to their ability to regulate synaptic function. For example, blockade and deletion of astrocytic Cx43 has been shown to impair fear memory consolidation and cause alterations in synaptic transmission and plasticity in rats (Pannasch et al., 2011; Stehberg et al., 2012).

There are limited studies on microglial connexins in the spinal cord. A study by Lee et al. (2005) using immunohistochemistry and triple labeling of Cx43, glial fibrillary acidic protein (GFAP; a marker of astrocytes) and OX-42 (a marker of microglia) showed that 1 week following SCI, Cx43 was colocalized with GFAP, rather than OX-42, suggesting that resting (ramified) and reactive (rounded phagocytic) microglia rarely express Cx43 in the spinal cord.

In oligodendrocytes, Cx29, Cx32, and Cx47 are expressed in regions of the corticospinal tract and are localized to oligodendrocytic cell bodies as well as abaxonal membranes of myelinated fibers, and these three connexins have been shown to participate in astrocytic/oligodendritic gap junctions (Kleopa et al., 2004; Li et al., 2004; Kamasawa et al., 2005). In examining astrocytic/oligodendritic interfaces, Nagy et al. (2001) observed astrocytic Cx43 and Cx30 staining at apposed oligodendrocyte somata in wild type mice. When Cx32 in oligodendrotcyes was knocked out, Cx30 disappeared, while Cx43 levels remain active, furthering the notion of a Cx43–Cx47 and a Cx30–Cx32 heterotypic astrocytic/oligodendritic coupling (Nagy et al., 2001). Similarly, Kamasawa et al. (2005) showed that on the oligodendrocytic side, a greater abundance of Cx47 compared to Cx32 corresponded to a greater level of Cx43 on the astrocytic side, further supporting the notion of Cx43–Cx47 and Cx30–Cx32 heterotypic channels as being the major components of astrocytic/oligodendritic gap junctions.

The most likely role of coupling between glial cells is the creation of the glial syncytium, which amongst other processes such as the propagation of Ca^2+^ waves and transfer of metabolites, plays a crucial role in spatially buffering increases in extracellular K^+^. In the CNS, a low extracellular K^+^ concentration is required for normal neuronal activity and thus pathological changes to connexin expressions are able to have a great impact on the balance of this system (Nagy and Rash, 2003; Rash, 2010). In addition, gap junction communication in glial cells is essential for normal central myelination. Indeed, mice deficient in both Cx32 and Cx47, which are expressed by oligodendrocytes, showed severe demyelinating phenotypes (Menichella et al., 2003; Odermatt et al., 2003). These double knockout mice developed gross tremors and tonic seizures, and died by the sixth postnatal week from profound abnormalities in central myelination. Abnormal changes included axons with thinly or absent myelinated sheaths, myelinated axons with markedly enlarged extracellular spaces separating the axon from its myelin sheath and occasional myelinated axons with enlarged collars of periaxonal oligodendrocyte cytoplasm (Menichella et al., 2003).

## ROLE OF GAP JUNCTIONS IN SPINAL CORD INJURY

Following a SCI, the initial impact leads to immediate hemorrhage and damage or disturbance to the neuronal elements of the spinal cord including severing of axon tracts and rapid cell death. The initial mechanical disruption is followed by multiple secondary cascades that cause further tissue loss and dysfunction. This secondary phase takes place during the first hours, days, or even months after the initial trauma. During this secondary phase, damage spreads to surrounding neuronal tissue, destroying gray and white matter in a process called the ‘bystander’ effect (Norenberg et al., 2004; Profyris et al., 2004). This encompasses glial cell responses such as astrogliosis and inflammatory cell activation, which can not only prevent neuronal repair, but also cause neuronal cell death. Cell death occurs via processes such as apoptosis, oxidative damage, and cytotoxic edema in astrocytes due to the buildup of glutamate and K^+^ (Del Bigio and Johnson, 1989; Crowe et al., 1997; Springer et al., 1999; Norenberg et al., 2004; Fitch and Silver, 2008).

In terms of specific connexins, an early study showed that following a mechanically induced SCI in rodents, alterations in astrocytic Cx43 occurred in gray and white matter of the spinal cord (Theriault et al., 1997). Reactive astrocytes displaying GFAP appeared within 1 day and reached maximum levels at 3 days in the area of injury and areas that were both adjacent to and farther away from the lesion epicenter. The authors concluded that during their transformation to reactive states, spinal cord astrocytes experience altered Cx43 expression as a direct result of traumatic SCI (Theriault et al., 1997). These results were replicated by Lee et al. (2005), who used a model of transected adult spinal injury and *in situ* hybridization showing that astrocytic Cx43 protein and mRNA levels were largely increased 4 h after injury, while there were no changes in Cx32 or Cx36. After 4 weeks, levels of astrocytic Cx43 were three times higher in the caudal stump than the rostral stump, especially adjacent to the injury site, suggesting that increases in Cx43 are related to glial responses to injured tissue (Lee et al., 2005). Several studies that used selective inhibition of Cx43 channels in *ex vivo* and *in vivo* models of SCI have shown that Cx43 plays a role in the spread of injury and its blockage leads to improved recovery (Cronin et al., 2008; Zhang et al., 2010).

While increases in Cx43 protein have been widely reported following SCI, this does not appear to be the case with all connexins. Following up on reports that Cx36 mRNA was down regulated at the site of injury during the first week after SCI (Lee et al., 2005), Yates et al. (2008) observed that 7 days after a complete T10 transection in rats, Cx36 protein levels decreased in the lumbar enlargement distant to the site of injury in association with decreased electrical coupling. While this downregulation lasted 14 days post-injury, Cx36 returned to control levels over 2–4 weeks. This coincided with the onset of hyperreflexia and was normalized following oral administration of the eugeroic agent, Modafinil (Yates et al., 2008). The changes in neuronal gap junction protein Cx36 below the level of the lesion suggest that disruption to electrical coupling contributes to hyperreflexia and spasticity following SCI (Yates et al., 2011).

Recently, studies have shifted attention from gap junctional activity to the role of undocked hemichannels following SCI. While it was previously thought that these unopposed hemichannels occurred in closed states, recent evidence shows that contrastingly, hemichannels are able to open independent of channel docking, in a regulated manner during specialized physiological events as well as unregulated opening during pathological events (Contreras et al., 2004; Retamal et al., 2006, 2007). These hemichannels form large cell pores in the cell membrane which, upon opening, allow the exchange of ions and small molecules between intra and extracellular environments. It was found that in addition to playing a traditional role in gap junctions, hexamers of Cx43 are inserted at astroglial membranes as large pore single hemichannels that are able to mediate direct cytosolic exchange with the extracellular space (Giaume et al., 2013).

Of the small molecule substances released by opened undocked hemichannels following SCI, particular attention has been directed at ATP (**Figure 1D**). ATP release following increased hemichannel expression has been demonstrated both *in vitro* and *in vivo*. The application of an acidic fibroblast growth factor-1 (FGF-1) is able to activate spinal astrocytes in culture, causing increased levels of ATP and the opening of connexin channels, in particular, Cx43 (Garré et al., 2010). FGF-1 acts on the FGF receptor, and its activation causes increased levels of cytoplasmic Ca^2+^ and the release of ATP from vesicles through the activation of phospholipase C (Bennett et al., 2012). When a FGF-1 inhibitor was applied following a weight drop SCI, this caused reduced ATP levels, accompanied by a reduction in inflammatory cell recruitment and lesion expansion (Bennett et al., 2012). A recent study using Cx43 KO mice specific for astrocytes showed that there was a reduction in ATP-induced inflammation, decreased macrophage and microglial recruitment, and an increased functional recovery following SCI (Huang et al., 2012). This study followed past observations that rats subjected to cord contusion have abnormally high and sustained levels of ATP release. By using bioluminescence imaging, levels of ATP in peritraumatic zones were found to be significantly higher than the center of injury, and this elevated ATP release persisted for up to 6 h following SCI. In addition, secondary enlargement of the lesion occurred with signs of inflammation that included increased expression of GFAP, and recruitment of activated microglia and macrophages (Wang et al., 2004). The link between ATP signaling and Cx43 was further strengthened in a recent study by Chever et al. (2014). It was found that when hippocampal CA1 pyramidal slices were treated with Gap26, a Cx43 hemichannel blocking peptide, there was a fivefold decrease in extracellular ATP concentration. However, when the slices were pretreated with ATP P2 receptor antagonists, Gap26 failed to induce changes in ATP concentration suggesting that basal excitatory synaptic transmissions are regulated by astroglial Cx43 hemichannels through ATP signaling (Chever et al., 2014). While there is still a need for further research to elucidate the exact mechanisms, past studies have been able to provide a framework by which Cx43 hemichannels contribute to SCI. Following SCI, increased opening of astrocytic Cx43 channels causes the uncontrolled release of ATP into the perilesion region and inflammatory cell recruitment such as macrophage and microglia, which in turn causes the increased secondary expansion of the lesion.

## ROLE OF GAP JUNCTIONS IN NEUROPATHIC PAIN FOLLOWING SPINAL CORD INJURY

As many as 80% of SCI patients will suffer some form of chronic pain; nociceptive (e.g., musculoskeletal pain, visceral pain), neuropathic pain (at-level SCI pain, below-level SCI pain), or other pain (Finnerup and Baastrup, 2012). Neuropathic pain is the more debilitating and difficult to treat. It is important to note that neuropathic pain represents a series of heterogeneous conditions including various painful peripheral neuropathies (e.g., traumatic nerve injury, chemotherapy-induced peripheral neuropathy, post-herpetic neuralgia) and central neuropathic pain, such as that occurring after SCI. However, despite the different etiologies and underlying mechanisms, the clinical manifestation of the pain is somewhat similar across the different neuropathic syndromes. Symptoms of both types of neuropathic pain include spontaneous pain presenting as burning, stinging, stabbing, or shooting sensations that is ongoing or intermittent; stimulus-evoked pain, which occurs in response to normally non-noxious (e.g., light touch) or noxious (e.g., cold, punctuate) stimuli; and abnormal sensations (e.g., paresthesia and dysesthesia). Neuropathic pain after SCI results from injury-induced neurochemical and neuroanatomical changes that contribute to maladaptive synaptic circuits and neuronal hyperexcitability in the spinal dorsal horn (Gwak and Hulsebosch, 2011). This pain has been reported as an important factor in decreased quality of life and has been shown to adversely impact function and daily routines in persons with SCI (Cairns et al., 1996; Siddall et al., 1999; Woolf and Mannion, 1999; Norenberg et al., 2004).

The conceptual basis of glial cells including astrocytes and microglia contributing to neuropathic pain is relatively new. The involvement of glial activation has been demonstrated in several chronic pain conditions, including inflammatory pain, peripheral, and central neuropathic pain (Wu et al., 2012). Activated glia can release ATP, excitatory amino acids, proinflammatory cytokines, and reactive oxygen species that in turn promote neuronal hyperexcitability in the dorsal horn of the spinal cord and contribute to neuropathic pain (Austin et al., 2012).

Glial activation has also been demonstrated in neuropathic pain following SCI (Hulsebosch et al., 2009). Several studies have demonstrated that rodents with SCI develop mechanical allodynia and thermal hyperalgesia, in association with astrocytic and microglial activation in segments that are uninjured and rostral to the initial injury site (Hains and Waxman, 2006; Peng et al., 2006; Carlton et al., 2009). Moreover, treatment with glial inhibitors, such as minocycline and propentofylline, reduced both mechanical allodynia and thermal hyperalgesia in the hind limbs of rats with below-level pain following SCI (Gwak et al., 2008; Gwak and Hulsebosch, 2009; Marchand et al., 2009).

Gap junctional activation via connexin channels has been proposed to play a role in glial cell activation, and thus neuropathic pain following SCI. Indeed, Roh et al. (2010) showed that in rats with T13 spinal cord hemisection the development of below-level neuropathic pain, including thermal hyperalgesia and mechanical allodynia, was attenuated following an intrathecal injection of carbenoxolone, a gap junction decoupler. In addition, daily treatment with carbenoxolone significantly reduced the SCI-induced increases in GFAP immunoreactivity and the phosphorylated NMDA receptor NR1 immunoreactivity in the dorsal horn of the spinal cord. These effects were observed when carbenoxolone was administered during the induction phase (0–5 days after SCI), but not during the maintenance phase (15–20 days after SCI), when pain is already established (Roh et al., 2010). This suggests there is a critical time window in which a treatment to block astrocytic gap junctions could be effective in stopping the development of SCI pain.

A recent study using a targeted deletion approach has shown that Cx43 is linked to the development of neuropathic pain following acute SCI (Chen et al., 2012). After a weight drop SCI, control wild type and Cx30 deleted mice developed persistent neuropathic pain lasting from 4 to 8 weeks, which presented as heat hyperalgesia and mechanical allodynia in the presence of increased levels of GFAP positive cells. However, in Cx43/Cx30 deleted mice, both the heat hyperalgesia and mechanical allodynia were prevented in addition to reduced levels of astrogliosis that continued up to 4 weeks. These findings indicate that Cx43 plays a significant role in the development of neuropathic pain following SCI.

## THERAPEUTIC APPLICATIONS

During the past two decades, intercellular communication via gap junctions has provided a novel pharmacological target for treating SCI. The proposed mechanism of gap junction inhibitors is to induce a conformational change in the gap junction protein to control the intercellular and metabolic signaling cascades, such as ATP, glutamate, and reactive oxygen species (Takeuchi et al., 2006; Ramachandran et al., 2007; Clarke et al., 2009). Currently, there are a number of non-specific gap junction blockers such as carbenoxolone, and the antimalarial mefloquine that blocks Cx36 gap junctions at low concentrations (3 uM), while at higher concentrations (30 uM), completely inhibits Cx43 as well as Cx26 and Cx32 in neuroblastoma cells (Cruikshank et al., 2004). In addition, there are novel gap junction hemichannel blockers, such as INI-0602, which can penetrate the blood–brain barrier, and is a proven and effective treatment in mice models of neurodegenerative diseases (Takeuchi et al., 2011). It was recently shown that administration of INI-0602 in mice with SCI resulted in reduced glutamate excitotoxicity by inhibiting microglial activation. This subsequently promoted locomotor recovery and suppressed glial scar formation by reducing secondary degeneration. Further, INI-0602 treatment blocked neutrophil infiltration, stimulated increase in anti-inflammatory cytokines and elevated brain-derived neurotrophic factor levels (Umebayashi et al., 2014). Although such non-specific connexin blockers may be promising therapeutic strategies in SCI, a problem with these drugs is that it is very difficult to control their actions on specific hemichannels and thus establish effective regulation. For instance, a recent study has demonstrated that intrathecal injection of carbenoxolone up to 5 days post-hemisection SCI attenuated the induction of neuropathic pain and the astrogliosis at the lesion edge, but did not significantly improve hindlimb locomotor function (Roh et al., 2010). These global inhibitors non-specifically block all types of gap junctions, affecting physiological functions, such as thermoregulation and angiogenesis (Betageri and Rogers, 1987; Hong et al., 2009). While the global gap junction blockers have confining or questionable control of lesion spread and astrogliosis following traumatic CNS injury, other tissues containing connexin gap junctions may also be adversely affected.

A more targeted approach using antisense oligodeoxynucleotides (AS-ODN) and connexin mimetic peptides has been developed to prevent connexin signaling. Mimetic peptides are short peptides designed to mimic sequences on extracellular loops of connexin proteins. They prevent connexin hemichannel docking, thus blocking gap junctional intercellular communication (Warner et al., 1995; Kwak and Jongsma, 1999; Boitano and Evans, 2000; Martin et al., 2005). Although the mechanism of action for connexin mimetic peptides has not been fully elucidated, it has been proposed that they may induce a conformational change in connexin hemichannels via connexin phosphorylation, leading to the closure of the uncoupled hemichannels without blocking existing gap junctions (Chaytor et al., 1997). In cultured rodent spinal cords, when a Cx43-mimetic peptide, peptide 5, was given at doses between 5 and 500 μm for 24 h, swelling was reduced by 50% and neuronal loss was prevented. Furthermore, peptide 5 was able to reduce GFAP levels for up to 4 days in *ex vivo* spinal cord segments following SCI (O’Carroll et al., 2008). This was further supported by a study that used an AS-ODN to suppress Cx43 following a compression SCI in rodents, showing that treatment with Cx43-ODN resulted in less swelling, structural distortion and decreased GFAP positive cells in spinal cord segments (Cronin et al., 2008). In a follow-up study, peptide 5 administered after a spinal cord contusion injury in rats showed reduced levels of proinflammatory cytokines tumor necrosis factor (TNF)-α and interleukin (IL)-1β, reduced GFAP staining in the area adjacent to the lesion epicenter, a reduction of neuronal loss 7 mm from the lesion epicenter as well as improved locomotor function (O’Carroll et al., 2013).

The application of connexin mimetic peptides has been widely studied in depressing immune responses (Neijssen et al., 2005; Matsue et al., 2006; Bopp et al., 2007), limiting cardiovascular damage (Wong et al., 2006; Shintani-Ishida et al., 2007), and inhibiting osteoclastic differentiation (Ilvesaro et al., 2001). Importantly, connexin mimetic peptides have the potential to be systemically delivered to target the lesion area of spinal cord in human patients, and offer a new hope to spinal cord-injured patients as an acute intervention. The regulation of Cx43 by AS-ODNs has been shown to not only protect neural cells after traumatic CNS injury, but also improve wound healing in many tissues, including cornea (Ratkay-Traub et al., 2001; Cursiefen et al., 2009; Grupcheva et al., 2012), skin (Coutinho et al., 2003; Qiu et al., 2003; Becker et al., 2012), skeletal muscle (Gorbe et al., 2006), cardiac muscle (Yasui et al., 2000), smooth muscle (Yeh et al., 1997; Liao and Duling, 2000), and vascular endothelium (Yeh et al., 2000; Kwak et al., 2001; Ormonde et al., 2012). Cx43 is upregulated at the wound edge of chronic wounds (Coutinho et al., 2003; Wang et al., 2007), and treatment with Cx43 AS-ODNs dampens the inflammatory response and decreases wound spread, resulting in reduced swelling and scarring and accelerated healing (Qiu et al., 2003; Coutinho et al., 2005; Mori et al., 2006). Significantly, several phase 1 or phase 2 clinical trials by CoDa Therapeutics, Inc. using the topical gel of Cx43 AS-ODNs (Nexagon^®^) have shown the treatment to be safe and tolerable in skin wounds (NCT00736593), venous leg ulcers (NCT00820196 and NCT01199588), diabetic foot ulcer (NCT01490879), and acute corneal wounds (NCT00654550). Thus, based on animal studies and the clinical use of connexin inhibitors in various conditions, drugs that inhibit specific connexins, such as Cx43 AS-ODNs or Cx43 mimetic peptides, hold great promise for the treatment of SCI.

## Conflict of Interest Statement

The authors declare that the research was conducted in the absence of any commercial or financial relationships that could be construed as a potential conflict of interest.
